# Takotsubo syndrome with apical thrombosis associated with hyperthyroidism crisis: a case report from high-altitude Tibet

**DOI:** 10.3389/fcvm.2025.1574352

**Published:** 2025-11-25

**Authors:** Jiaheng Zhang, Lixue Yin, Qingfeng Zhang

**Affiliations:** 1 School of Medicine, University of Electronic Science and Technology of China, Chengdu, China; 2 Ultrasound Medicine and Computational Cardiology Key Laboratory of Sichuan Province, Sichuan Provincial People’s Hospital, University of Electronic Science and Technology of China, Chengdu, China

**Keywords:** thrombosis, hyperthyroidism, takotsubo syndrome, echocardiography, high altitude

## Abstract

**Background:**

Catecholamine surge is considered the primary trigger of Takotsubo syndrome(TTS), but its pathophysiological mechanisms remain incompletely understood. Cases of TTS with intracardiac thrombus induced by thyrotoxicosis in the setting of high-altitude hypoxia are particularly rare.

**Case summary:**

A 65-year-old woman living at 3,200 m was admitted with abdominal and chest pain. On admission, her BP was 108/68 mmHg, HR 135 bpm, Temp 37.8°C, and SpO₂ 95% on room air. Hemoglobin was 168 g/L, consistent with chronic high-altitude adaptation. Coronary angiography at the local hospital showed mild stenosis of the left anterior descending artery. She presented with tachycardia, marked ST-segment elevation, and elevated troponin T and B-type natriuretic peptide (BNP) levels. The ST-segment elevation gradually resolved during hospitalization, arguing against acute myocardial infarction. However, markedly elevated thyroid hormone and thyrotropin receptor antibody levels were indicative of a thyroid storm. Transthoracic echocardiography (TTE) revealed apical hypokinesia and ballooning accompanied by an apical thrombus. Myocardial contrast echocardiography (MCE) indicated delayed and sparse perfusion in the apical segment, while Magnetic resonance imaging (MRI) ruled out remote myocardial injury. Mildly elevated myocardial enzymes and rapid resolution of ST-segment elevation supported the diagnosis of Takotsubo cardiomyopathy. Thyrotoxicosis may have enhanced myocardial sensitivity to catecholamines, predisposing to stress-related injury. Chronic high-altitude hypoxia can further increase sympathetic activity and impair coronary microcirculation. The patient was treated with *β*-blockers, antithyroid agents, and anticoagulation, along with supportive therapy targeting oxidative stress, which was followed by regression of the apical thrombus and improvement in cardiac function.

**Conclusion:**

The combined effects of severe thyrotoxicosis and chronic high-altitude hypoxia may induce TTS and its related complications by enhancing sympathetic activity and catecholamine responsiveness.

## 1 Introduction

TTS is characterized by transient systolic dysfunction of the apical and middle left ventricle in the absence of obstructive coronary artery disease. Possible mechanisms include sympathetic stimulation, catecholamines, coronary spasm, and microvascular dysfunction (1). Evidence linking thyroid disease to TTS supports the well-established theory that sympathetic overactivity and hyperthyroidism can contribute to the onset of the condition (2). This case specifically examines a patient experiencing hyperthyroidism crisis in the Tibetan mountainous region.

## 2 Case presentation

**Figure 1:**
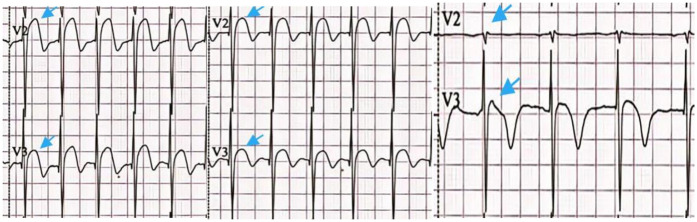
ECG of the first/second/fourth day of hospitalization. ST elevation gradually decreased as indicated by the blue arrows.

A 65-year-old female was admitted to the hospital due to recurrent lower abdominal pain associated with a hyperthyroidism crisis. She is a Tibetan patient from an altitude of approximately 3,200 m. The patient had a history of hypertension, which was adequately controlled with amlodipine 5 mg daily. She denied any previous use of antithyroid or thyroid-related medications and had no prior history of diabetes or known coronary artery disease. On admission, BP 108/68 mmHg, HR 135 bpm, Temp 37.8 °C.The blood gas analysis results were as follows: pH 7.44, pCO₂ 31 mmHg, pO₂ 36 mmHg, HCO₃^−^ 21.1 mmol/L, and SO₂ 92%. (3,200 m),and hemoglobin was 168 g/L, these findings indicate mild respiratory alkalosis and moderate hypoxemia, consistent with chronic adaptation to high-altitude hypoxia. Since the onset of the disease, The patient was alert but mildly agitated. The electrocardiogram showed extensive ST-segment elevation, with 0.4 mV elevation in leads V1-V3 and 0.2 mV elevation in leads V4-V5. The ST elevation gradually decreased with the length of hospital stay (Figure 1). The myocardial enzyme increase was not apparent, troponin I: 88.4 ng/L (cutoff value <15.6 ng/L), creatine kinase within normal range. A computed tomography scan suggested a gallbladder stone, but no other obvious abnormality existed. The values of thyroid-stimulating hormone(TSH), TT3, TT4, FT3, FT4, and Anti-TSHR were 0.001 mIU/L (0.35–0.94), 0.53 nmol/L (0.88–2.44), 250.73 nmol/L (62.8–150.8), 29.02 nmol/L (2.6–5.7), 45.14 nmol/L (9.0–19.0), and 16.32 IU/L (<1.22), respectively. The combination of persistent sinus tachycardia (135 bpm), mild agitation, and low-grade fever without evidence of infection or acute coronary syndrome raised suspicion of thyrotoxicosis. A Burch–Wartofsky score of 65 (temperature 5, tachycardia 25, gastrointestinal/hepatic 20, central nervous system-CNS 5, hypoxic stress 10) supported the diagnosis of thyroid storm.

**Figure 2:**
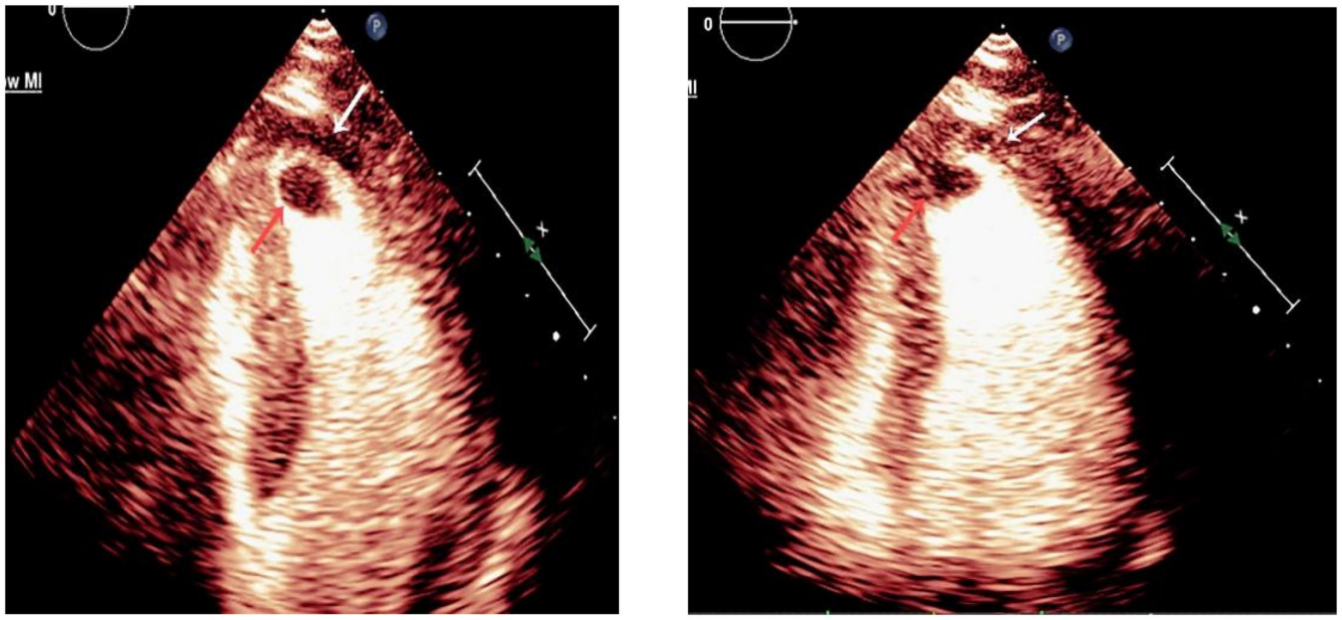
Left: MCE revealed filling defect in the apical region, along with reduced myocardial perfusion. The orange arrow indicates the thrombus, while the white arrow highlights the impaired myocardial perfusion. Right: Improved myocardial perfusion with thrombus reduction. The orange arrow highlights the thrombus, while the white arrow marks the myocardial perfusion.

**Figure 3:**
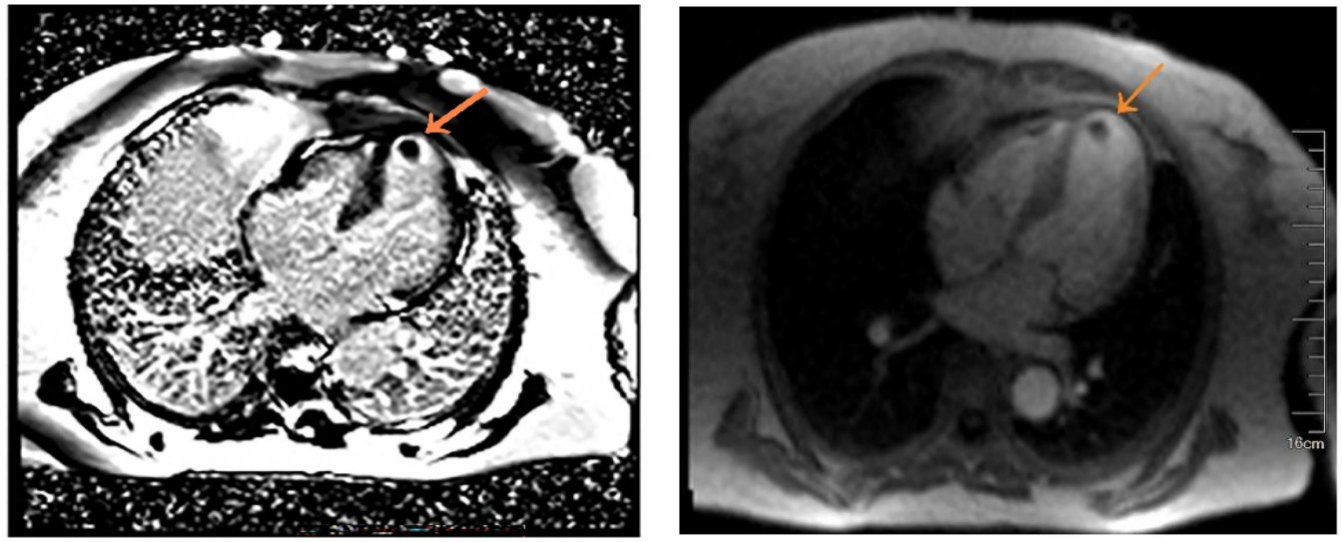
MRI showing an apical thrombus,and first pass perfusion was normal as indicated by the orange arrow.

**Figure 4:**
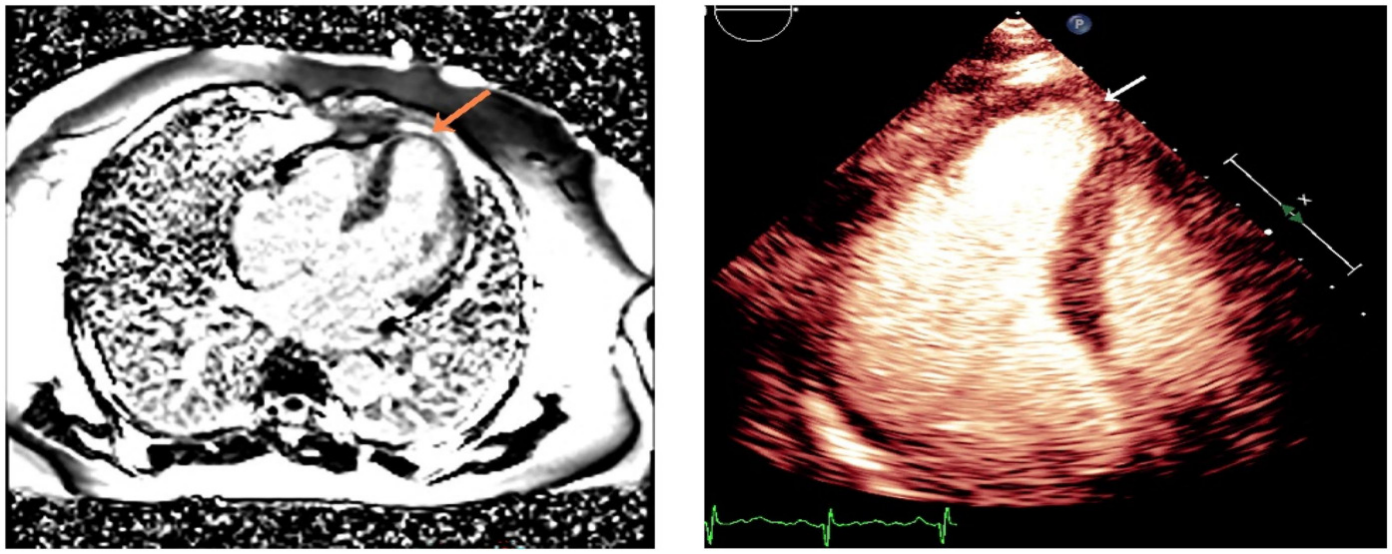
Three months later, MRI and MCE revealed the disappearance of the apical thrombus, with MCE showing significant improvement in myocardial perfusion. The orange arrow indicates the disappearance of the thrombus,while the white arrow highlights indicates the improved myocardial perfusion.

Sequential multimodality imaging including abdominal CT, myocardial contrast echocardiography (MCE), and cardiac magnetic resonance imaging (MRI) was performed to exclude abdominal pathology and to further characterize the cardiac abnormalities. Transthoracic echocardiography (TTE) revealed hypokinesia of the mid and apical segments with characteristic apical ballooning. And the absence of coronary obstruction strongly supported the diagnosis of thyroid storm-related Takotsubo syndrome rather than ischemic heart disease. MCE further showed delayed and sparse myocardial perfusion in the apical region, and an apical thrombus measuring 28 × 19 mm was identified (Figure 2). The patient was transferred to the cardiac intensive care unit, where her symptoms gradually improved. One week later, follow-up TTE showed improved wall motion, with the thrombus reduced to 20 × 11 mm and ejection fraction increased to 57% (Figure 2). MRI subsequently demonstrated normal first-pass perfusion and no late gadolinium enhancement, ruling out remote myocardial injury (Figure 3). The therapeutic regimen included propranolol (0.25 mg) for adrenergic control and inhibition of peripheral T4-T3 conversion, low-dose irbesartan (20 mg) for afterload reduction and myocardial recovery during the acute phase, rivaroxaban (20 mg) for anticoagulation, and methimazole (5 mg) for thyroid suppression. At the three-month follow-up, left ventricular systolic function had normalized (EF: 65%) (Table 1), the apical thrombus had disappeared (Figure 4), and thyroid function tests showed complete biochemical recovery.

**Table 1:** Serial clinical, laboratory, and imaging findings.

Parameter	Admission	1 week	3 months
HR (bpm)	135	92	80
LVEF (%)	45	57	65
LVOT-VTI (cm)	14	18	20
BNP (pg/mL)	420	80	65
Troponin T (ng/L)	88.4	20.3	<15
Urine output (mL/kg/h)	1.1	1.2	1
FT3 (pmol/L)	29.0	7.8	4.8
FT4 (pmol/L)	45.1	21	15.2
TSH (mIU/L)	0.001	0.08	0.42

## 3 Discussion

Although awareness of TTS has increased among clinicians worldwide, its pathophysiological basis remains only partially understood. Accumulating evidence suggests that excessive catecholamine exposure induces direct myocardial toxicity and metabolic stunning, whereas concomitant microvascular dysfunction may compromise myocardial perfusion and further exacerbate the transient contractile impairment characteristic of this syndrome (3). Initially considered a reversible heart failure syndrome with self-limiting clinical course, TTS is now recognized to potentially lead to serious complications such as ventricular arrhythmias, thromboembolism, and cardiogenic shock (4). There are documented associations with thyroid disease, supporting the widely accepted pathophysiological theory that sympathetic hyperactivity and hyperthyroidism can promote TTS (5). However, the precise frequency of this association remains unknown. This case represents a unique constellation of a definitive thyroid storm, chronic high-altitude exposure, and a large apical thrombus, providing a distinctive clinical model to explore the synergistic interplay among extreme endocrine, sympathetic, and environmental stressors in the pathogenesis and complications of TTS. While recent studies have primarily focused on the epidemiology, diagnosis, management, and prognosis of TTS (6), its precise pathophysiology remains incompletely understood. In our case, the sequential use of multimodal imaging and biochemical evaluation ensured diagnostic accuracy and minimized the risk of misclassification. The coexistence of severe thyrotoxicosis and chronic high-altitude hypoxia likely exerted a synergistic effect on sympathetic activation (7). The thyroid storm acted as an acute trigger for catecholamine surge in a myocardium already sensitized by chronic hypoxic stress, ultimately leading to catecholamine-mediated myocardial stunning and microvascular dysfunction (8). Moreover, differential diagnosis in this case included acute coronary syndrome, myocarditis, and hyperthyroid cardiomyopathy. The absence of obstructive coronary lesions, normal myocardial perfusion on MRI, and the transient pattern of regional wall motion abnormalities distinguished TTS from these conditions.

How do catecholamines interact with the thyroid gland? Catecholamines interact with the thyroid gland through complex interplay involving the adrenergic axis. Elevated levels of thyroid hormones can lead to hyperchronotropic and inotropic responses to catecholamines. This effect is partly mediated by the upregulation of *β*-adrenergic receptors in various tissues, including the heart (9). Excessive thyroid hormone levels can trigger sympathetic overactivation, leading to acute heart failure. Optimal management involves combination of antithyroid therapy and *β*-blocker therapy, which can rapidly improve left ventricular systolic function. The patient received supportive therapy with receptor blockers for TTS, along with antithyroid drugs and antithrombotic therapy for thyrotoxicosis. It is worth noting that propranolol, used in TTS treatment, lowers heart rate and inhibits T4-to-T3 conversion, providing both cardiovascular and metabolic benefits in thyrotoxicosis. The convergence of a hypercoagulable state from thyroid storm, coupled with potential chronic hypoxemia-induced endothelial dysfunction at high altitude, likely created a perfect storm that predisposed this individual to thrombus formation (10). MCE revealed sparse apical perfusion, which improved after one week, indicating microcirculatory injury during early stage. Coronary microvascular dysfunction remains an important pathophysiological link between sympathetic overactivity and transient ventricular dysfunction (11, 12). The heightened hypoxic susceptibility in Tibetan populations suggests oxidative stress as a potential therapeutic target (13). This case emphasizes the need to elucidate how thyroid function, high-altitude adaptation, and cardiac vulnerability interact in the development of TTS.

## 4 Conclusion

In thyrotoxic patients, adrenergic receptor upregulation and catecholamine excess may trigger TTS, while high-altitude hypoxia can further amplify sympathetic activation and microvascular dysfunction. Achieving hemodynamic stability requires prompt use of methimazole with alpha- and beta-blockers, and identifying underlying triggers is essential for optimal management.

## Data Availability

The original contributions presented in the study are included in the article/Supplementary Material, further inquiries can be directed to the corresponding author.
